# Adolescents’ Experiences of Participating in Sensitive Research: A Scoping Review of Qualitative Studies

**DOI:** 10.1177/15248380211069072

**Published:** 2022-01-19

**Authors:** Lakshmi Neelakantan, Deborah Fry, Lani Florian, Franziska Meinck

**Affiliations:** 1 Department of Psychiatry, 6396 University of Oxford, Oxford, UK; 2 Moray House School of Education and Sport, 128598 The University of Edinburgh, Edinburgh, UK; 3 School of Social and Political Science, 151027 University of Edinburgh, Edinburgh, UK; 4 OPTENTIA, Faculty of Health Sciences, North-West University, Vanderbijlpark, South Africa

**Keywords:** violence exposure, child abuse, trauma, qualitative research, youth violence

## Abstract

Despite an increasing emphasis on adolescents’ participation rights, there are concerns about their participation in research on sensitive topics, such as trauma and violence. This review reports findings of a scoping review that examined the nature and extent of qualitative studies conducted with adolescents about their experiences of participating in research on sensitive topics. Studies were identified by searching electronic databases and grey literature and reported on qualitative and mixed-methods studies eliciting adolescents’ experiences of participating in research on sensitive topics. Seventeen (17) studies were included after screening 4426 records. The scoping review revealed significant adolescent benefits from participation, relating to positive emotions, skill acquisition and enhanced self-efficacy and interpersonal relationships. To a lesser extent, participants also experienced burdens relating to negative emotions, concerns about confidentiality and privacy and inconvenience of participation, which were mitigated by careful attention to research design and researcher engagement and training. Participants shared insights into their motivation to participate, and factors that impacted their experiences of research, such as ethical considerations, including consent procedures, safety and connection in research, study procedures and documentation and researcher characteristics. There were tangible benefits and some burdens involved in adolescents’ participation in sensitive research. This review considers implications for research and practice, such as the need to regularly publish findings of consultations, assessing caregiver consent requirements, obtaining adolescent views on study documents and measures and building on existing research, differentiated by age, gender and dis/ability status, especially in diverse and under-represented regions.

Participation, broadly conceptualized as being consulted and making decisions, is intended to advance adolescents’ right to be involved in all decisions that affect them and have their views considered, including in research activities (Alderson, 2008; Lansdown & UNICEF Innocenti Research Centre, 2018). Respect for children’s freedom of expression and their views is enshrined in Articles 12 and 13 of the United Nations Convention on the Rights of the Child (UNICEF, 1989), and several frameworks have conceptualized this complex idea (Hart, 1992; Lundy, 2007; Shier, 2001). There is an increasing emphasis on involving children and adolescents in research that is relevant to them (Rahimzadeh et al., 2015), with supporting arguments citing pedagogical benefits (what children can learn from the experience), political potential (children’s ability to change social policy and exercise rights), epistemological context (children being able to enhance current understanding), consumer benefits (the potential for improved value and design of services) and protectionist concerns (developing respectful dialogue to enhance child protection) (Tisdall et al., 2009).

However, there are concerns around involving children and adolescents in research on sensitive topics such as child abuse, sexual health and trauma, due to their age (Alderson & Morrow, 2011) and perceived lack of competence (Bradbury-Jones & Taylor, 2015), meaning that children are assumed to lack capacity and maturity to participate in research meaningfully (Lundy et al., 2011). Researchers report numerous challenges involved in gaining ethical approval and doing research on sensitive topics with both children and adolescents (Graham et al., 2015), such as institutional processes unfamiliar with the topic and design (Hays et al., 2003), a focus on children and adolescents’ ‘vulnerability’, sometimes at the cost of closing down their participation rights, and adult gatekeepers limiting access to young people (Angell et al., 2010; Skelton, 2008), meaning valuable perspectives may be lost (Hildebrand et al., 2015). For instance, research conducted on children’s needs around domestic violence still relied on adult proxies, such as parents, policymakers or professionals (Noble-carr et al., 2019). While this may vary across contexts, researchers generally also have a threshold duty to act as mandated reporters in research projects and may be obligated to report instances of suspected child abuse to the appropriate authorities (Allen, 2009).

Further, gatekeepers at multiple levels, namely guardians, domain (school or other organisation), organisational and institutional levels, may facilitate or limit access to children and adolescents for research studies, based on their own views and priorities (Kay, 2019). Researchers working on sensitive topics are accustomed to a high degree of justified scrutiny, but this can, at times, become an extended bureaucratic process that excludes children and adolescents from participating (McAreavey & Das, 2013; Schelbe et al., 2015). Requirements for caregiver consent, which are commonly mandated by ethics committees, can also impede participation and in some cases, lead to systematic exclusion of marginalized youth, for example, LGBTQ+ populations (Schelbe et al., 2015).

It is increasingly acknowledged that adolescence is a distinct period in human development, when adolescents begin to engage actively with their rights, form important connections with peers and seek to influence decisions that impact them (Lansdown & UNICEF Innocenti Research Centre, 2018). This means that adolescent perspectives of sensitive research are distinct and valuable and may vary from children and adult perspectives. While adolescents’ views of sensitive research are critical in creating better institutional structures that appropriately balance protection concerns with participation rights, the evidence on this topic is mixed. Reviews examining sensitive topics, have focused on adults’ experiences (Jaffe et al., 2015), adolescents’ views of services (Brodie et al., 2016) or adolescents’ experiences of participating in research on specific topics such as violence and abuse (McClinton Appollis et al., 2017; McClinton Appollis, Lund, De Vries, & Mathews, 2015). There is little evidence on adolescents’ own voices and in-depth perspectives on participating in research on a wider range of sensitive topics. Thus, the purpose of the current review is to highlight existing research on adolescents’ perspectives of participating in research on sensitive topics. We focus on adolescents’ experiences across global settings to identify key common lessons from studies to inform future research, practice and policy. To our knowledge, this is the first review that prioritizes adolescents’ experiences of sensitive research where their own voices are centred.

## Methodology

### Objectives

This article reviews the literature on adolescents’ experiences of participating in sensitive research topics using a scoping review approach. Scoping reviews “aim to map rapidly the key concepts underpinning a research area and the main sources and types of evidence available” (Arksey & O’Malley, 2005). The purpose of this scoping review is to locate and summarise the limited research on adolescents’ experiences of participating in research on sensitive topics. We followed the framework proposed by Arksey and O’Malley (2005) in its approach, complemented by the guidelines laid out by Levac et al. (2010). We report study methods and findings in line with the Preferred Reporting Items for Systematic reviews and Meta-Analyses extension for Scoping Reviews (PRISMA-ScR) (Tricco et al., 2018). This is detailed in Appendix A. We registered the protocol on Joanna Briggs Institute Database of Systematic Reviews and Implementation Reports and Open Science Framework Registries (Neelakantan, 2019a; 2019b) on September 12th and October 15th, 2019, respectively.

### Search Strategy

The following databases were searched up to December 2020: PsycINFO, Medline, PubMed, EMBASE, CINAHL and ProQuest Dissertations and Theses. Scoping searches on Google Scholar and grey literature on the World Bank, World Health Organisation (WHO), United Nations Population Fund (UNFPA) and UNICEF websites, were also searched. Reference lists of all identified reports and articles were searched, and requests for information were circulated through a weekly newsletter sent out by the Sexual Violence Research Initiative (SVRI), an email list that reaches several thousand researchers in the field of violence prevention globally. Searches were limited to studies published in English. A full search strategy undertaken with MEDLINE is detailed in Appendix B. This search strategy was used in all databases with necessary adjustments made for truncations, wildcards and Boolean operators.

### Eligibility Criteria

Qualitative or mixed-method studies focussing on adolescents’ experiences of participating in research on sensitive topics were included. Qualitative research was prioritised as this best amplifies adolescents’ voices and complex perspectives. Adolescents were defined as individuals aged 10–19 years, reflecting UNICEF/WHO definitions (WHO., 2021).

This review considered topics proposed by Lee and Lee (2012) and van Meter (2001) as ‘sensitive research’, namely HIV/AIDS, violence against children, drug use and substance abuse, sexuality and sex-related topics, including LGBTQ issues. This review also considered mental illness and suicide to be sensitive topics due to widespread stigma associated with these topics and their consistent links to other sensitive topics listed above (Casale et al., 2019; Jackson-Best & Edwards, 2018; Kaushik et al., 2016).

Studies exploring children and adults’ perspectives were excluded. In studies where there was a mix of adolescent and non-adolescent participants, the study was included if the majority of participants were adolescents. Reviews were excluded, but their reference lists were searched for suitable studies.

### Screening, Data Charting and Synthesis

Screening was done using Rayyan, a review software (Ouzzani et al., 2016). After removing duplicates, one reviewer (LN) screened 4426 studies for eligibility. Of these, 4381 titles and abstracts were identified using electronic databases and 45 studies from other sources. One reviewer (LN) screened 172 full-text studies and included 17 studies in the review. Most studies were excluded because they focused on the views of caregivers and other adults rather than prioritising adolescent perspectives, used only quantitative methods to assess adolescent views, presented researcher reflections on participant experiences or otherwise focused on experiences of undergoing violence or trauma, and not on experiences of research.

One reviewer (LN) extracted the study characteristics, findings, recommendations and limitations of each study and entered them into a customised table (see Table 1). All authors contributed to the narrative synthesis of included studies by comparing, discussing and consolidating included studies into categories, and identifying relationships between key findings to fulfil review aims (Levac et al., 2010). Any disagreements were resolved among the authors through discussion. Findings were organized according to topic areas the included studies themselves focused on, namely motivations to participate, experiences of research participation and outcomes of participating in research.

**Table 1.** Characteristics of included studies.

Author (Year), Location	Participants and Methods	Findings of Motivation	Findings of Research Interactions	Findings of Outcomes	Study Recommendations	Study Limitations
Chappell et al. (2014), South Africa	3 youth aged 15–20 years (2 female, 1 male). Qualitative (group discussions)		Youth perceived their relationship with the principal researcher to be shifting throughout the research process. This relationship shifted from one where the adult was perceived to be in charge, to one where everyone was on a more equal footing. This helped the youth recognise their own agency and ownership	Taking part in research enabled participants to create other identities and speak out. Youth co-researcher offered valuable perspectives on knowledge co-construction, learnt about the topic and themselves, and gained several practical life skills. The dialogic space offered by research enabled internal dialogue and new views of oneself, and shared dialogue with others	The research process enacts permeable relationships between the principal and co-researchers. Both these groups undergo changes in self-positions that were mutually informed through interactions. These interactions benefit the youth as well as the research study	Focus is on co-researchers, but this is an admittedly small group of youth
Cluver et al. (2020), South Africa	65 adolescents aged 10–18 years. Qualitative (adolescent advisory groups, individual interviews, anonymous postboxes, feedback on social media, WhatsApp groups)		Adolescents emphasized the importance of safety and openness in initiating and sustaining engagement, while noting safety itself could take time to be established. A stigma-free space was important, as were fun and enjoyable activities. Co-designing workshops and feeling connected to the research was critical. Personal relationships with the researchers were important but boundaries between researchers and adolescents shifted with continued engagement	Adolescents contributed extensively to the design and implementation of large and complex studies at several levels of the research lifecycle, from refining research questions, and selecting study settings to designing study materials and questionnaires and conducting fieldwork. Adolescent personal development was an important aspect of their participation. This was valued and requested	Advisory groups are feasible and with the investment of time and effort, can help reduce power differences. Safe, beneficial, and non-stigmatising advisory groups can bring about frank engagement, which is fundamental to research. Recognising adolescents' expertise and using their experiences to improve outcomes for other young people are ways of ensuring meaningful engagement	Gender split for participants not provided. Data collected not as part of a separate research study but over years of both formal and informal engagement
Cody (2017), Albania, Bulgaria, and England	47 adolescents and young people aged 11–25 years, with at least 27 adolescents aged between 11-18 years. At least 39 female participants. Qualitative (group consultation workshops with various activities)	Young people in ongoing situations of abuse, who were facing ongoing threats of safety, or with past negative experiences, especially if they were recent, may be hesitant to participate. Reliving trauma, stigma, embarrassment, and fears of identification were challenges in participation	Young people emphasized the importance of creating spaces to talk about sexual violence, sex, and relationships in general. Young people feel comfortable with other young people, so training and other initiatives on sexual violence could be led by young people. Dialogue with young people was needed to ensure that language used in prevention initiatives was appropriate. Young people identified key principles for participation, including several facilitator characteristics. Incentives, opportunities for skill development, ensuring balanced time investment, and ensuring that participation is fun, creative and safe were important while planning initiatives	Gaining new skills and knowledge was valued, as was helping other young people. Young people gained knowledge about sexual violence and felt satisfied that their voices would be heard	Young people are able to assess the risks of participation and highlighted the importance of being ‘ready’ to participate, which necessitates an ongoing process of interaction. They value that the work was meaningful, organised well, and was ‘official’. Young people’s involvement in sexual violence prevention initiatives could lend great value	The majority of participants were female. Findings were not presented separately for those under 19 years and over. Focus of study was not young people’s experiences of research but more broadly on prevention efforts. Multi-site study, therefore, some meanings may have been lost in translation
Coors and Raymond (2009), USA	9 adolescents aged 15–18 years (all male). Qualitative (focus group discussions)		Adolescents were concerned about the stigma associated with participation in substance use genetic research and that collected material may be used improperly by the criminal justice system. They were less concerned about the confidentiality of their DNA.		Participants may not be familiar with risks of participating in psychiatric gene research relating to substance use disorder, even after an extensive consent procedure. More easily accessible information is needed at subsequent points in the study	Focus of study was not experiences of participation, but on substance use disorder research and ethical concerns were addressed very briefly in topic guide and findings. Small sample
Demkowicz et al. (2020), England	Two studies: In first, 68 adolescents aged 10–16 years; in second, adolescents aged 8–16 years (M 11.88 SD 2.06, 45 female, 23 male). Mixed-method (qualitative interviews and focus groups used in qualitative component)		Adolescents were both highly concerned with and reassured by anonymity offered by the study, including the physical spaces in which the study was conducted. They emphasized the importance of having an option to talk to someone afterwards. Understanding the purpose of the study and what exactly was expected of them was very important. Adolescents consistently highlighted the structure wording, format, ease of responding to the items and response options, and length of completion as important in their experiences	Adolescents valued the effect participation had on being able to release emotions, reflected on their lives and experiences, took stock of positive experiences, and gained new skills, such as in emotional self-regulation	Undertake piloting of all measurement tools, including cognitive interviewing, and adapt instruments to ensure accessibility. Written information sheets may not always be read, so present key information in an accessible manner and remind the importance of anonymity throughout, including at the end, with resources clearly signposted. Work with gatekeepers to clarify that participation is voluntary and make clear that participants can skip any items they want. Ensure private spaces where participants can answer	Sample comprised of participants who volunteered which might result in bias. Little demographic information so difficult to unpack variations among the sample. Timing of data collection may have resulted in loss of emotionally salient experiences
Devries et al. (2015), Uganda	40 adolescents aged 12–14 years (22 female, 18 male). Mixed-method (qualitative structured, open-ended interviews used in qualitative component)		Interviewer characteristics, training, and trust were critical in adolescents' decision to disclose experiences of violence. The main factor in their decision to disclose was an expectation of help. Adolescents experienced relief at being able to discuss their experiences and did not perceive the interview as traumatic. This was not universal, and participants otherwise expressed feeling scared about their information being passed on, recalling the pain of abuse, and feeling ‘bad, then good'		Adolescents did not perceive the research interview as traumatic and reported positive experiences, however this is borne out by careful interviewer training and full debriefing. Consider the possibility of retaliatory violence if in future surveys the subject of the study becomes known to the wider community, although this risk was low in this study. While an expectation of help was important in adolescents' experiences, studies would do well to clarify that participants may not receive a direct immediate benefit from participation	Part of a larger study examining referrals undertaken in research on violence against children and experiences of adolescents with the child protection system, and experiences of adolescents with research were reported within this
Edwards et al. (2016), USA	204 adolescents aged 13–18 years (M 15.56 SD 1.32, 117 male, 85 female). Mixed-method (open-ended questions on survey used in qualitative component)			3 (1.5%) and 12 adolescents (5.9%) reported regretting participation and feeling upset. 99 adolescents (48.5%) reported experiencing benefits from participation. Upset feelings were linked to peers’ opinions (especially regarding bystander non-action), disliking the recall of negative memories, and discomfort with questions. Benefits extended to knowledge, awareness, and skills gained, and a broad range of interpersonal gains	Burdens significantly outweighed by benefits associated with participation. Consent forms and information sheets may include information about research-related upset feelings and researchers may also include debriefing procedures. Such debriefing may include factual information on domestic violence and safety skills	The study used single-item, brief questions to assess reactions to research participation and additionally only assessed immediate reactions to participation
Hasking et al. (2015), Australia	1973 adolescents aged 12–18 years (M 13.89, SD 0.97). Mixed-method (open-ended questions on survey used in qualitative component)			Benefits included understanding and reflection, knowing that help was available, helping others, having fun, self-expression, and getting out of class. Burdens included boredom, negative emotions, such as feeling upset, recalling past experiences, worries about confidentiality, and worries about relationships with others	Research with adolescents promotes help seeking behaviours and improving the prognosis for young people who may be distressed. Recent negative life events may cause participants to feel upset, so resources must be made available to young people. Commitments to confidentiality need to be reiterated as participants were not always sure how researchers handled their data	Two items assessed reactions to participating in research. Part of a mixed method study exploring quantitative outcomes on research participation. Low response rates which may have resulted in bias
Houghton (2015), Scotland	8 young people aged 15–19 years. Qualitative (individual interviews, focus group discussions, group activities, and debriefs)		Young people advanced the conceptualization of participation from the three Cs (consent, confidentiality, child protection) and three Ds (danger, distress, disclosure) to include the three Es as well (enjoyment, empowerment, and emancipation). The three Es reflected the importance of building trust, recognising that young people had an individual, expert voice, and ensuring real power that resulted in real impact for abused children and young people. Confidentiality and privacy were critical, as was the possibility of distress which was however viewed as inevitable and something that needed to be managed. Researcher specialist training was important in navigating difficult reactions in the study		Young people co-developed an ethical framework which recognised and accounted for their emotional, social, and moral capacities. Recognising children’s agency is critical, while ensuring that participation is safe and part of their therapeutic experience. It is even more critical for those who had experienced abuse to be involved. A presumption of empowerment was necessary and young people want to know that they could change other children’s lives	Small group of young people who participated in the ethics study, within a larger study exploring perspectives on domestic abuse policy
Lockwood et al. (2018), England	594 adolescents aged 13–15 years (M 13.5 years, SD 0.61, 51% male, 49% female). Mixed-method (open-ended question and doodle activity used in qualitative component)			Benefits included understanding and reflection, altruism, and research itself as an enjoyable and interesting activity. Burdens included experiencing negative emotions and finding the research boring or irrelevant. Participants also critically engaged with the research process and offered suggestions for its improvement	Mood calibration doodle page at the end of the survey increased engagement and allowed participants to calm emotions. Recently experienced negative events, such as self-harm, were usually accompanied by negative events, so researchers may plan for this. Participant reactions are varied and coupled positive and negative ratings, suggesting that distress is not necessarily a negative outcome and benefits and risks may co-occur	One item used to assess responses to participation. Part of a larger mixed-method study using quantitative methods to assess participant reactions
Moore et al. (2020), Australia	35 adolescents aged 9–16 years (17 males, 18 females). Mixed-method (focus group discussions used in qualitative component)	Motivating factors for deciding to participate included benefits for others, catharsis, a sense of duty or obligation, the prospect of developing new skills, contributing to service improvement, connecting with peers, financial incentives, consulting with others or being influenced by those inviting them to participate, and credibility of the research project/researchers. Adolescents also considered the sensitivity of topics that the research may be asking about and deliberated how this might be defined			Internal and external motivations for participants were quite important, but dependent on the context and participants. Adult stakeholders thus need to be aware of sophisticated decision-making that children and adolescents undertake, and researchers need to be aware of the motivations for participation. Establishing relationships with participants and other stakeholders would enable accurate information to be conveyed and decisions made	Sample comprises of those participants whose caregivers also consented, which could lead to bias. Limited information on demographics which makes it difficult to transfer findings to other contexts and sub-groups
Notley et al. (2015), England	13 adolescents aged 16–23 years (9 male, 4 female). Mixed-method (semi-structured interviews used in qualitative component)		Adolescents appreciated a practical and measured approach to all research procedures. They also perceived the research to be broadly acceptable, and sensitive aspects of the research were deemed acceptable due to researcher training and reassurance. Overall presentation, and information about research was generally considered to be important	Disclosure was experienced as positive and therapeutic. Altruism was another important benefit. Engagement with the intervention had benefits apart from the intervention itself. The research was also perceived as challenging, especially with feeling apprehensive and worried, but these reduced with time	Presentation of assessment measures and interpersonal skills of the researcher are important factors influencing acceptability of study procedures. Careful piloting and assessment of recruitment and information sheets also recommended	Participants may have assumed the interviews were part of the wider trial and their responses subject to social desirability bias
Renold et al. (2008), Wales	6 adolescents aged 10–20 years (3 female, 3 male). Qualitative (field notes, observations, and interactions)		Attempting to engage with young people on reflections and ethical processes directly did not often work but embedding ‘ethical talk’ in everyday research interactions (i.e. indirectly) was much more successful. Participants also entered and exited states of participation, becoming ‘participant’ and then ‘non-participant’, for example, by not engaging when asked how they felt about being in a research project, and participants editing and sharing bits of data they wanted to with the researcher		Participation may be more usefully enacted and examined as ‘ethical talk’ in everyday fieldwork, rather than as a fixed entity. Participation is also always in negotiation and extends beyond the binary as give and take. Developing personalized ethical protocols may be helpful, as will not viewing informed consent as a one-off event. Researchers may also engage in continual reflexive practice to examine ethics in the field	Small sample of young people. Findings move between researcher-reported and young-person reported
Robbins et al. (2012), Australia	58 adolescents aged 10–15 years (54% female, 46% male). Mixed method (focus group discussions used in qualitative component)	Adolescents had mixed feelings before research began, viewing research as broadly positive, but also suspicious of longitudinal research on health and sexuality. Both external (peer and parental influences, incentives) and internal influences (altruism, making friends, seeking new experiences, learning about themselves, and study acknowledgement) were important in their decision to participate		Adolescents had positive feelings about their participation and viewed focus groups as engaging methods	Peer recruitments are helpful in aiding motivation to participate. Detailed explanations of research purpose and methods can increase interest in participation. Establishing goodwill and building on it throughout aids participant engagement	Participants were from independent or private schools, so were from a specific socioeconomic background, which may lead to bias. Peer opinions may have led to group consensus
Vander Laenen (2009), Belgium	160 adolescents aged 12–21 years (29 female, 131 male). Qualitative (focus group discussions)		Adolescents were highly distrustful of researchers at the beginning, although trust was built gradually over time. Their interactions were also marked by a feeling of powerlessness and scepticism that their views would be taken seriously, which they articulated was important to them		Young people negotiate their agency within the balance of power in specific situations, which may manifest as negative or positive interactions. Researchers may expect resistance especially where highly sensitive topics such as drug use are discussed. Decide on methods and spaces where participants can freely express opinions, ensure that no authority figures are present, and validate participants' opinions by offering feedback and taking results back to them	Highly skewed sample, majority males. There were significant dropouts from the study, but for non-research purposes. However, this may have resulted in differing findings across time
Wallace-Henry (2015), South Africa	28 adolescents aged 9–17 years. Qualitative (focus group discussions, participatory activities, and journals)			Children and adolescents engaged with Hart’s model of participation and suggested that children self-advocate on their own behalf, with the support of their parents when necessary. Mechanisms to facilitate children and adolescents' contributions were necessary. Barriers to participation included the perception of children as morally and cognitively incompetent. Adolescents appreciated the contribution to knowledge they had made	Adolescents are active agents who provide useful insights into issues that concern them and beliefs and factors that exist in their subculture. Supportive environments must exist to enable them to speak out	Limited information on outcomes, brief findings provided
Whittington (2019), England	103 young people aged 13–15 years, with 75 young people under the age of 18 years (71 female, 31 male, 1 non-binary). Qualitative (group sessions)			Participants gained various skills, including in critical thinking and reflection, awareness about the topics covered in research, self-protection, and in co-producing research data. Participants also gained interpersonal skills	Active and reflexive ethics can enable a more democratic research process which acknowledges power relations and prioritises participant autonomy. Gaining vocabulary, skills, and competence to navigate challenging situations can contribute to safeguarding. Safeguarding procedures should seek to manage rather than avoid risk. Obtaining young people’s perspectives before the design and methodology is finalised would be helpful, along with a focus on participant rights rather than only vulnerabilities	Researcher-reported findings are prominent, relative to adolescent reported views

**Figure 1.**
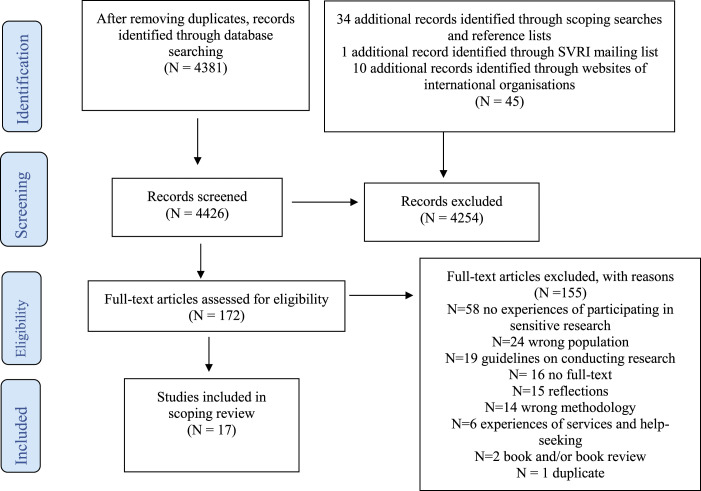
PRISMA flow diagram for scoping review.

Details of the search and screening process are provided in a PRISMA Flow Diagram in Figure 1. Quality appraisal of included studies was not undertaken as this is not generally recommended in scoping reviews, whose aim is to map the available evidence rather than to provide a synthesised and clinically meaningful answer to a question (Peters et al., 2020). This is consistent with other scoping reviews carried out (Arksey & O’Malley, 2005; Lal et al., 2012; Levac et al., 2010; Schmidt et al., 2018; Wilson et al., 2015).

**Appendix A:** Preferred Reporting Items for Systematic Reviews and Meta-Analyses Extension for Scoping Reviews (PRISMA-ScR) Checklist

Section	Item	PRISMA-ScR Checklist Item	Reported on Page #
TITLE			
Title	1	Identify the report as a scoping review	Title Page
ABSTRACT			
Structured summary	2	Provide a structured summary that includes (as applicable): background, objectives, eligibility criteria, sources of evidence, charting methods, results, and conclusions that relate to the review questions and objectives	Abstract
INTRODUCTION			
Rationale	3	Describe the rationale for the review in the context of what is already known. Explain why the review questions/objectives lend themselves to a scoping review approach	Page 1–3
Objectives	4	Provide an explicit statement of the questions and objectives being addressed with reference to their key elements (e.g. population or participants, concepts, and context) or other relevant key elements used to conceptualize the review questions and/or objectives	Page 3
METHODS			
Protocol and registration	5	Indicate whether a review protocol exists; state if and where it can be accessed (e.g. a web address); and if available, provide registration information, including the registration number	Page 3
Eligibility criteria	6	Specify characteristics of the sources of evidence used as eligibility criteria (e.g. years considered, language, and publication status), and provide a rationale	Page 4,5
Information sources*	7	Describe all information sources in the search (e.g. databases with dates of coverage and contact with authors to identify additional sources), as well as the date the most recent search was executed	Page 4
Search	8	Present the full electronic search strategy for at least 1 database, including any limits used, such that it could be repeated	Page 4–5, Appendix B
Selection of sources of evidence†	9	State the process for selecting sources of evidence (i.e. screening and eligibility) included in the scoping review	Page 5–6
Data charting process‡	10	Describe the methods of charting data from the included sources of evidence (e.g. calibrated forms or forms that have been tested by the team before their use, and whether data charting was done independently or in duplicate) and any processes for obtaining and confirming data from investigators	Page 5–6
Data items	11	List and define all variables for which data were sought and any assumptions and simplifications made	Not applicable
Critical appraisal of individual sources of evidence§	12	If done, provide a rationale for conducting a critical appraisal of included sources of evidence; describe the methods used and how this information was used in any data synthesis (if appropriate)	Not applicable
Synthesis of results	13	Describe the methods of handling and summarizing the data that were charted	Page 5–6
RESULTS			
Selection of sources of evidence	14	Give numbers of sources of evidence screened, assessed for eligibility, and included in the review, with reasons for exclusions at each stage, ideally using a flow diagram	Page 5
Characteristics of sources of evidence	15	For each source of evidence, present characteristics for which data were charted and provide the citations	Page 6–7, Table 1
Critical appraisal within sources of evidence	16	If done, present data on critical appraisal of included sources of evidence (see item 12)	Not applicable
Results of individual sources of evidence	17	For each included source of evidence, present the relevant data that were charted that relate to the review questions and objectives	Not applicable
Synthesis of results	18	Summarize and/or present the charting results as they relate to the review questions and objectives	Pages 7–15
DISCUSSION			
Summary of evidence	19	Summarize the main results (including an overview of concepts, themes, and types of evidence available), link to the review questions and objectives, and consider the relevance to key groups	Pages 15–17
Limitations	20	Discuss the limitations of the scoping review process	Pages 20, 21
Conclusions	21	Provide a general interpretation of the results with respect to the review questions and objectives, as well as potential implications and/or next steps	Page 21
FUNDING			
Funding	22	Describe sources of funding for the included sources of evidence, as well as sources of funding for the scoping review. Describe the role of the funders of the scoping review	Title page

From: Tricco AC, Lillie E, Zarin W, O'Brien KK, Colquhoun H, Levac D, et al. PRISMA Extension for Scoping Reviews (PRISMAScR): Checklist and Explanation. Ann Intern Med. 2018; 169:467–473. doi: 10.7326/M18-0850.

**Appendix B:** 

No	Sample Search Strategy: PsycINFO, MEDLINE and Embase on Ovid
1	adolescen* or teen* or youth* or young* or child*
2	views or experience* or attitudes or risk or benefit or harm or recommendation or perceptions or participat* or cogniti* or ethic*
3	abuse or neglect or violen* or trauma or adversity or drug* or sex* or disability or illness
4	2 and 3
5	Sensitive adj research
6	4 or 5
7	Qualitati* or mixed-meth*
8	1 and 6 and 7

## Results

### Characteristics of Included Studies

A total of 17 studies were included in this scoping review. Of the 17 studies, 4 were from England, 3 from Australia, 3 from South Africa, 2 from the United States, 1 from Uganda, 1 from Scotland, 1 from Wales and 1 from Belgium. One study was conducted in multiple contexts, namely Albania, Bulgaria and England. The studies were published between 2008 and 2020.

Studies did not consistently report the gender make-up of participants, but of studies which reported this, 5 studies comprised of mostly female participants (Chappell et al., 2014; Cody, 2017; Demkowicz et al., 2020; Robbins et al., 2012; Whittington, 2019), 4 studies included mostly male participants (Coors & Raymond, 2009; Edwards et al., 2016; Notley et al., 2015; Vander Laenen, 2009) and 4 studies included a roughly equal number of male and female participants (Devries et al., 2015; Lockwood et al., 2018; Moore et al., 2020; Renold et al., 2008). Of the 17 studies, 9 were standalone qualitative studies (Chappell et al., 2014; Cluver et al., 2020; Cody, 2017; Coors & Raymond, 2009; Houghton, 2015; Renold et al., 2008; Vander Laenen, 2009; Wallace-Henry, 2015; Whittington, 2019), while 8 were qualitative studies within a larger mixed-method study (Demkowicz et al., 2020; Devries et al., 2015; Edwards et al., 2016; Hasking et al., 2015; Lockwood et al., 2018; Moore et al., 2020; Notley et al., 2015; Robbins et al., 2012).

Study samples ranged from 3 participants (Chappell et al., 2014) to 1973 participants (Hasking et al., 2015) with the larger studies employing mixed methods. Participants were aged 10–19 years old in most studies; however, some participants were older than 19 years in six studies (Chappell et al., 2014; Cody, 2017; Notley et al., 2015; Renold et al., 2008; Vander Laenen, 2009; Whittington, 2019).

Several studies focused on more than one sensitive topic such as HIV/AIDS, adolescent pregnancy, parenting, and violence (Cluver et al., 2020), non-suicidal self-injury, psychological distress, abuse, and suicidal behaviour (Hasking et al., 2015) and sensitive issues as a broad category (Moore et al., 2020). Others focused on sexuality, sexual consent, and sexual and reproductive health (Chappell et al., 2014; Robbins et al., 2012; Whittington, 2019), young people in care (Renold et al., 2008), violence and abuse (Cody, 2017; Devries et al., 2015; Edwards et al., 2016; Houghton, 2015; Wallace-Henry, 2015), substance use (Coors & Raymond, 2009; Vander Laenen, 2009), psychological difficulties and mental health (Demkowicz et al., 2020; Notley et al., 2015) and self-harm (Lockwood et al., 2018).

## Findings

### Adolescents’ Motivations to Participate

Five studies discussed findings on adolescents’ motivations for participating in sensitive research (Cody, 2017; Devries et al., 2015; Moore et al., 2020; Notley et al., 2015; Robbins et al., 2012), which we categorized as external and internal motivations.

While younger adolescents identified external motivations such as financial incentives as vital, older adolescents viewed them as a ‘bonus’ rather than a critical motivator for participation (Robbins et al., 2012). However, other adolescents highlighted that financial incentives might be viewed as a ‘bribe’ or diminish the sense of altruism participants might have, suggesting views on these could vary across age groups and contexts (Moore et al., 2020). They also noted that financial incentives must be age-sensitive, for example, phone cards, music downloads, movie passes, rewards for the participant’s family and sponsored school excursions (Robbins et al., 2012). Non-financial incentives also featured prominently, for example, a certificate or public announcement, developing new skills, being afforded the opportunity to discuss important topics, learning from others, being exposed to new experiences, interacting with their peers and improving local services (Moore et al., 2020; Robbins et al., 2012).

Other external motivations included relational factors such as parental encouragement, peer influence (Robbins et al., 2012), and other adults who invited adolescents to participate, such as a teacher or staff member (Moore et al., 2020). The perceived credibility of the researcher was also an important factor, reflected in markers such as working for a ‘legitimate’ organisation or university and being recruited through a school or a trusted organisation as opposed to social media (Moore et al., 2020).

Internal reasons for participating were consistent across studies, namely altruism, that is, selfless concern for the wellbeing of others, making social contributions, discoveries and new friends, informing adolescents about certain practises or programmes and helping adults better appreciate what young people think and feel (Moore et al., 2020; Robbins et al., 2012). Altruism was a significant motivator even in control groups in randomised studies (Notley et al., 2015) and adolescents reported they would be more likely to participate in a study, even if it might cause some discomfort or be time consuming (Moore et al., 2020). An expectation of receiving help, as might be communicated by the phrasing in consent forms, was also an important consideration (Devries et al., 2015).

Other reasons included a belief in a duty or obligation to participate in research, especially if adolescents held leadership positions in school, although this was not uniformly observed (Moore et al., 2020). Participants also highlighted catharsis or ‘getting things off your chest’ as an important reason, especially for those who had previously undergone negative experiences, although this was not preferred in a group setting (Moore et al., 2020). Conversely, having had past negative life experiences, ongoing abuse or possible threats to safety could hinder participation (Cody, 2017). For group-based research, the size of the group was also an important consideration for adolescents, with smaller groups preferred (Cody, 2017).

### Adolescents’ Experiences of Research Interactions

#### Ethical Considerations

Adolescents conceptualized ethical procedures, by expanding Mullender et al. (2002)’s mnemonic of three Cs (consent, confidentiality, and child protection), and three Ds (danger, distress, and disclosure), to include children’s position and agency, expressed by the three Es (enjoyment, empowerment, and emancipation) (Houghton, 2015). Importantly, adolescents emphasized that participation should be fun, creative, and comfortable, in addition to being safe (Cluver et al., 2020; Cody, 2017). Activities that reduced adult-adolescent power discrepancies were particularly helpful, such as co-developing engaging survey questionnaires designed as teen magazines, painting t-shirts, board-games, campfires, movie nights, graffiti sessions and talent shows (Cluver et al., 2020).

For facilitating disclosure, adolescents highlighted the importance of peer support, trusted relationships with researchers, researcher support and empathy and a full explanation of study procedures (Devries et al., 2015; Houghton, 2015; Notley et al., 2015). Disclosure, however, could also be a complex process, as adolescents reported feeling scared about their information being passed on (and therefore being reluctant to disclose), recalling the pain of their original abuse and feeling “bad, then good” (Devries et al., 2015).

Issues with confidentiality, anonymity and protection of privacy were concerning for participants in several studies (Cody, 2017; Coors & Raymond, 2009; Demkowicz et al., 2020; Devries et al., 2015; Houghton, 2015; Notley et al., 2015). Adolescents’ concerns around confidentiality also depended on the information under question, for example, while adolescents participating in substance abuse disorder research were not concerned about the confidentiality of retained DNA, they were concerned that the results of psychiatric research may be inadvertently used within the criminal justice system (Coors & Raymond, 2009).

However, the promise of confidentiality and privacy was critical in adolescents’ decisions to disclose ongoing experiences of violence and seek help (Demkowicz et al., 2020; Devries et al., 2015). Suggestions to address these concerns in the context of a school-based study included allowing study completion in smaller groups rather than full classes, ensuring pupils did not sit directly next to one another, providing private spaces in schools to individually complete study measures and sharing web links with students to complete at home (Demkowicz et al., 2020).

***Findings on consent procedures*.** The above findings on confidentiality and privacy concerns suggest that participants may have been unclear about other aspects of studies as well, which has important implications for the ongoing nature of informed consent (Hasking et al., 2015). Participant responses suggested that they had not fully understood the information sheet and consent forms used in studies (Demkowicz et al., 2020; Hasking et al., 2015), and this impacted their experiences of participation, including their understanding of confidentiality and privacy. In addition, adolescents expressed being unsure of how long the study would take (Notley et al., 2015), whether they could stop answering questions, and if they could skip certain items, which suggests that at the time of completion, they did not have the level of information needed to participate without concerns and questions about ethical considerations (Demkowicz et al., 2020).

### Safety and Connection in Evolving Research Interactions

Evidence suggests that interactions in sensitive research were a shifting and evolving process, undergoing changes with time (Chappell et al., 2014; Cluver et al., 2020). At the beginning, research interactions were likely to resemble power relations in adult–adolescent relationships, but as the study progressed, adolescent co-researchers began to view their relationship with the principal researcher on a much more equal footing, which was reflected in the adolescent co-researchers’ decision to suggest strategies for how the research could be conducted (Chappell et al., 2014). As relationships evolved and deepened, adolescents also looked to researchers for help and assistance with topics not related to research, for example, financial and educational support, reproductive health advice, school and professional development (Cluver et al., 2020).

Safety and connection were important in creating research spaces for adolescent participants in sensitive research (Cluver et al., 2020; Cody, 2017; Notley et al., 2015; Vander Laenen, 2009). Adolescents appreciated that spaces are not often available for adolescents to discuss about sexual violence, sex and relationships, and get information on these topics (Cody, 2017). Research spaces free of stigma were particularly valued by adolescents who had experienced HIV/AIDS in their family or lives, especially in the form of Whatsapp group chats which members relied on for informal communication and support (Cluver et al., 2020). It was important for adolescents to feel connected to the research, by being afforded opportunities to co-design advisory groups and understand the aims of workshops, for example, HIV/AIDS status disclosures were addressed by adolescent-led ground rules signed by each member.

Some adolescents began participating in research with fear, apprehension, worry or a lack of trust due to a habitual sense of being guarded or cautious in their communities (Cluver et al., 2020), believing the researcher may share research interactions with other adults (Vander Laenen, 2009), and concerns that a psychological intervention may be painful (Notley et al., 2015). Helpful strategies in establishing trust included the following: researchers introducing themselves and sharing their own stories, establishing a personal connection with participants, guaranteeing anonymity repeatedly, keeping in mind the individuality of each participant, answering questions participants might have about their lives and continuing group talk among participants, allowing the researcher to fade into the background (Cluver et al., 2020; Vander Laenen, 2009).

### Research Procedures, Questionnaires and Documents

Research procedures, questionnaires and documents used were important in adolescents’ experiences of participation (Cluver et al., 2020; Cody, 2017; Demkowicz et al., 2020; Devries et al., 2015; Houghton, 2015; Notley et al., 2015; Vander Laenen, 2009). While the range of constructs and questions used in a study provided variety and allowed adolescents to think deeply about their feelings, this also meant that some questions were difficult to understand, or relate to, especially those which were hypothetical or scenario-based (in this case, one which measured stigma) (Demkowicz et al., 2020).

Items were perceived as unclear due to vague wording, double-barrelled questions, unfamiliar words, the temporal nature of questions, such as when participants had to consider the last month or the last 2 weeks, and the contexts participants considered, such as schools or homes (Demkowicz et al., 2020). Likert scales also elicited mixed responses, with some adolescents finding that the distinctions between anchors were difficult to understand. Adolescents had mixed views on including items which were sensitive, or elicited personal information (Demkowicz et al., 2020; Notley et al., 2015). They found these acceptable when they were reassured by the researcher that they did not have to answer them (Notley et al., 2015), and despite some discomfort with sensitive items, they were not perceived as impacting their overall experience (Demkowicz et al., 2020).

### Researcher Characteristics

Favourable researcher characteristics included researcher skill and training (Devries et al., 2015; Houghton, 2015), a person-centred approach (Notley et al., 2015) and developing a personal connection with participants (Cluver et al., 2020). In group settings, adolescents connecting with each other were powerful, as they found it easy and comfortable to engage with peers, especially if they were from similar cultural and linguistic contexts (Cluver et al., 2020; Cody, 2017). In general, adolescents valued researchers listening to young people (Vander Laenen, 2009), allowing them to lead, being non-judgemental, giving them time, using ordinary and accessible language, involving young people from various backgrounds (Cody, 2017) and being genuinely warm and sensitive to high stigmatization or gossip (Cluver et al., 2020).

### Outcomes of Research Participation

#### Benefits and Burdens of Participation

Several studies supported the emotional benefits of participation, with adolescents appreciating research as a space for reflection on their life and experiences, an opportunity for catharsis and offloading emotions and experiencing gratitude and a sense of perspective about their life experiences (Demkowicz et al., 2020; Devries et al., 2015; Hasking et al., 2015; Lockwood et al., 2018). Adolescents also reported gaining skills, such as improved knowledge and critical thinking about the topics covered in research (Cody, 2017; Edwards et al., 2016; Whittington, 2019), confidence (Cluver et al., 2020; Cody, 2017), awareness of job-seeking procedures (Cluver et al., 2020) and improved emotional self-regulation (Demkowicz et al., 2020). Other benefits included better interpersonal skills to deal with, among others, family challenges and peer interactions (Cluver et al., 2020; Edwards et al., 2016; Whittington, 2019). 

However, participants also highlighted several burdens such as feeling upset and worried during participation (Devries et al., 2015; Edwards et al., 2016; Hasking et al., 2015; Lockwood et al., 2018; Notley et al., 2015). Feeling upset was driven by several reasons, including recalling past life experiences (Devries et al., 2015; Edwards et al., 2016; Hasking et al., 2015; Lockwood et al., 2018), worrying about their information being passed on to others (Devries et al., 2015; Lockwood et al., 2018), disliking the questions themselves (Edwards et al., 2016; Lockwood et al., 2018) and contemplating other adolescents’ difficult life experiences (Hasking et al., 2015).

Adolescents greatly appreciated the contribution to knowledge they made and the opportunities for altruism that research afforded (Cody, 2017; Edwards et al., 2016; Hasking et al., 2015; Lockwood et al., 2018; Vander Laenen, 2009; Wallace-Henry, 2015). Opportunities for seeking and receiving help (Devries et al., 2015; Hasking et al., 2015), and enjoying new experiences, such as exploring natural environments safely were perceived as beneficial (Cluver et al., 2020). Several studies also highlighted the benefit of enjoying and engaging with research processes as a separate benefit apart from the study itself (Cluver et al., 2020; Edwards et al., 2016; Hasking et al., 2015; Lockwood et al., 2018; Notley et al., 2015; Robbins et al., 2012).

Other burdens included perceiving the research as boring, irrelevant or inconvenient (Hasking et al., 2015; Lockwood et al., 2018). Burdens may be mitigated by full debriefing and careful and extensive interviewer training (Devries et al., 2015; Edwards et al., 2016; Houghton (2015). The above burdens experienced by participation were not universal across studies; the identified benefits of participation were more numerous, measured by the number of participants who identified benefits as opposed to burdens. Across studies, greater numbers of participants identified their participation as beneficial rather than burdensome.

### Adolescent Contribution to Research

Adolescents contributed extensively to the co-creation of research, including designing several large-scale longitudinal and randomised studies on violence, HIV/AIDS and adolescent pregnancy on the African continent, helped refine research questions and select study settings, designed remote engagement strategies during the Covid19 pandemic, co-designed adolescent-friendly quantitative and qualitative research tools, co-designed training for fieldwork staff and shared views on feasibility of research methods (Cluver et al., 2020). Involving adolescents in the data analysis yielded insights into sex and relationships of disabled youth that may not have been available to the principal researcher alone (Chappell et al., 2014), and language that may be used to describe sexual violence in prevention initiatives (Cody, 2017). These activities not only created highly tailored and useful policy guidelines that were directed by adolescents themselves, but also built confidence among participants (Cluver et al., 2020).

## Discussion

This review highlighted key findings on adolescents experiencing numerous benefits and some burdens while participating in sensitive research. Moreover, this review detailed how research procedures such as information provided about the study, clarity of measures used, ethical concerns around anonymity and confidentiality and characteristics of researchers played an important role in how adolescents experienced participation. Finally, this review highlighted important conceptualizations of ethical procedures advanced by adolescents themselves, which emphasized enjoyment, empowerment and emancipation, in addition to more conventional concerns around confidentiality, consent, child protection, danger, distress and disclosure.

The findings on benefits and burdens are consistent with researchers’ reflections on doing research on sensitive topics with children and adolescents (Radford et al., 2017) and quantitative studies on adolescents’ reactions to sensitive research (Finkelhor et al., 2014; McClinton Appollis et al., 2017; Polihronis et al., 2020; Ybarra et al., 2009). These findings also align with evidence on adolescent participation in non-sensitive research, where adolescents found participation to be beneficial, valued incentives and potential for altruism and preferred to be consulted on participating in research (Crane & Broome, 2017). Ultimately, these findings demonstrate the value of well-designed studies which enhance the benefits of participation to the best extent possible, while minimizing burdens (Alderson & Morrow, 2011; Graham et al., 2015; Kyegombe et al., 2019; O’Reilly & Parker, 2014; Shaw et al., 2011).

Moreover, this review builds a nuanced understanding of adolescents’ motivations to participate and experiences of participation, which is a new contribution to current literature. This review has shown how adolescents considered internal factors, such as catharsis, altruism, feelings of duty, past negative experiences and an opportunity for reflection in their decision making on participation. It also described external factors, such as the sensitivity of the topic, financial and non-financial incentives and receiving help, as motivation to participate in research on sensitive topics. Adolescents’ feelings of relief and catharsis during participation align with evidence on adult emotions in sensitive research (Aroussi, 2019).

We did find some variation in findings across studies conducted in the same country, for example, such as views of adolescents in Australia on financial incentives to participate, suggesting that such preferences may naturally vary across adolescents of different ages, genders, socioeconomic status and location, and therefore, where possible, research should seek adolescent preferences on such topics. Across countries, while findings varied on specific adolescent preferences for consent procedures and questionnaires and confidentiality concerns around specific types of information, higher-level findings did not vary significantly, and remained consistent for experiences and outcomes of participation.

Returning to the arguments for involving children and adolescents in research, this review shows that the benefits of adolescent participation in sensitive research are consistent across most domains identified by Tisdall et al. (2009), namely adolescents learn from the experience, they are able to change policy and exercise rights, their views produce better research and improved services, and enhance child protection efforts. Equally, however, adolescents reported several emotional and practical burdens of participation, which are important to address in future studies, by incorporating information research-related upset in information sheets and debriefing procedures that address common burdens experienced during participation (Edwards et al., 2016). While more steps to enhance participant safety and wellbeing throughout participation is needed, these findings underscore the numerous benefits adolescents gain from participating in research on sensitive topics.

Our findings further underscore the importance of involving adolescents in all research pertaining to them, but especially on sensitive topics. Speaking specifically to institutional barriers that researchers navigate to conduct research on sensitive topics, these findings demonstrate that in seeking to protect adolescents, we must not inadvertently overlook the numerous benefits that adolescent participation brings to themselves and the research as a whole. We hope that these findings encourage stakeholders, gatekeepers and institutional ethics committees to balance adolescents’ right to be protected from harm with their right to participate and benefit from research.

### Recommendations

#### Incorporate Adolescent Perspectives into Sensitive Research Studies

Several studies highlighted the salience of ethical procedures in adolescents’ experiences of participation. Reframing ethical concerns to include enjoyment and fun was important to adolescents and is important to note for future research studies (Cluver et al., 2020; Cody, 2017; Houghton, 2015; Lockwood et al., 2018). Of particular interest were persistent adolescent concerns regarding issues of anonymity, confidentiality and privacy, despite these having been addressed in information sheets. There were also issues with participants knowing whether they could skip answering questions and if they could stop answering at any time. These issues suggest that the notion of ongoing informed consent is not always addressed in a way that participants can understand and more engaging and memorable ways of communicating study information are needed.

Given ongoing challenges that researchers face in gaining ethical approval for research on sensitive topics, it would also be valuable to explore how existing requirements for caregiver or guardian consent could be modified to balance legal requirements with adolescents’ preferences. Studies show that by the age of 14, adolescents’ understanding of health research and participant rights is similar to adults (Macapagal et al., 2017). While this study did not explore adolescent preferences on caregiver consent for adolescent participation in research, research studies on informed consent with sexual and gender minority in the USA recommend that self-consent is prioritised and caregiver consent is waived as it is a significant barrier to participation and such consent could itself violate the privacy and confidentiality of adolescents (Fisher et al., 2016). More research is therefore needed with adolescents in various countries and age groups on informed consent requirements to generate appropriate recommendations.

### Build on Existing Research

To build on these findings and obtain a fuller picture of adolescent experiences of participation, more research on adolescent perspectives needs to be undertaken consistently. Given that guidelines put forth by funding agencies emphasise participation (National Institutes for Health, 2017), it is recommended that adolescents are consulted much more routinely on their views and how participation in sensitive research could be made meaningful and valuable. Research studies may already be consulting adolescents in pilot or post-completion studies, but these are rarely published, and we recommend that these findings are published regularly, so these perspectives can inform other studies as well. Moreover, there is little understanding of how outcomes relating to participation could be measured consistently across studies, which is an important area of future research. Emerging work on conceptual frameworks to measure outcomes of adolescent participation is a useful starting point (Lansdown & UNICEF Innocenti Research Centre, 2018).

Adolescent perspectives on specific study procedures, such as information sheets, clarity of measures used, format and mode of instruments and interpretations of questions and response options are quite rare. This suggests that there needs to be further research in employing specific study procedures with adolescents. When research is undertaken with adolescents, their views on participation are not consistently elicited and published, and if they are, their perspectives are not necessarily applied to the analysis of data. Further, our understanding of what might constitute sensitive topics are not informed by how adolescents might define such terms, so foundational research needs to be undertaken to explore how these concepts are defined and understood by adolescents themselves. Finally, while several studies reported the impact of research activities on adolescents, very few studies reported the impact that adolescent participation had on research activities. This is an important outcome that must be reported in future research endeavours.

### Ensure that Adolescent Voices are Heard from All Contexts

While this review found a few studies from low- and middle-income countries which elucidated such perspectives in rich detail, they were typically outweighed by evidence emerging from high-income countries (e.g. the UK) and certain middle-income countries, such as South Africa. Interestingly, there were no studies on adolescents’ experiences from Asia, where more than half of all adolescents globally live (UNICEF, 2019). One could posit from these findings that research on adolescents’ experiences appears particularly localised to certain regions and countries. Children and adolescents in low- and middle-income countries experience several co-occurring burdens (Meinck et al., 2015), so perspectives from diverse and under-researched regions are all the more important in informing recommendations for conducting sensitive research.

### Limitations

This review has a number of limitations. First, this was a scoping and not a systematic review, and therefore does not claim to be exhaustive, although a comprehensive search strategy was adopted, and efforts were made to capture a wide range of studies. Second, this review only considered materials available in English. This understandably results in gaps generated in relation to studies conducted and published in other regions. Third, there are limitations in the existing evidence base that forms the base for this scoping review, as much of what has been published on children and adolescents’ participation in sensitive research emerges from high-income countries (Know Violence in Childhood, 2017). While efforts were made to locate literature from low- and middle-income countries and studies from other contexts were found, the findings in this review emanate from studies predominantly conducted in high-income countries. It is possible that such questions were explored in the form of pilot or post-completion studies conducted in low- and middle-income countries but were not published. It is surprising that there is limited research on this topic as it is challenging to obtain ethical approval for conducting large-scale research on sensitive research topics, and so qualitative evidence on adolescents’ experiences would be especially valuable in highlighting the benefits and burdens of such research accurately. Finally, since included studies predominantly had small samples and did not always present findings by relevant sample characteristics such as age, gender, socio-economic status, we were unable to systematically disaggregate findings according to these categories.

## Conclusion

This review has identified the numerous benefits adolescents derived from participating in research on sensitive topics, alongside important burdens which must be addressed in future research studies. Adolescents also displayed complex and sophisticated decision-making in deciding to participate in research on sensitive topics and made several critical contributions to conducting and disseminating sensitive research on topics relevant to their lives. This review has, however, identified an urgent need for greater and more consistent adolescent involvement in sensitive research, which extends to piloting and testing instruments, measures and study materials, and incorporating adolescent perspectives in analysing data and generating findings. This may require developing frameworks for measuring adolescent participation in research studies and developing meanings of sensitive topics, both of which must be informed by adolescent voices.
